# The effect of fidget spinners on fine motor control

**DOI:** 10.1038/s41598-018-21529-0

**Published:** 2018-02-16

**Authors:** Erez James Cohen, Riccardo Bravi, Diego Minciacchi

**Affiliations:** Department of Experimental and Clinical Medicine, Physiological Sciences Section, University of Florence, Florence, Italy

## Abstract

Fidgeting, defined as the generation of small movements through nervousness or impatience, is one of cardinal characteristic of ADHD. While fidgeting is, by definition, a motor experience still nothing is known about the effects of fidgeting on motor control. Some forms of fidgeting involve also the manipulation of external objects which, through repetition, may become automatic and second nature. Both repetition and practice are important for the acquisition of motor skills and, therefore, it is plausible that the repetitive manipulation of objects may influence motor control and performance. As such, fidget spinners, by being diffuse and prone to repetitive usage, may represent interesting tool for improving motor control. In this study we examine the effect of fidget spinners on fine motor control, evaluated by a spiral-tracing task. We show that the use of fidget spinner indeed seems to have a favorable effect on fine motor control, at least in the short term, although this effect does not seem to be in any way inherent to fidget spinners themselves as much as to object manipulation in general. However, due to their widespread usage, fidget spinner may have the advantage of being an enjoyable means for improving fine motor control.

## Introduction

**Figure 1:**
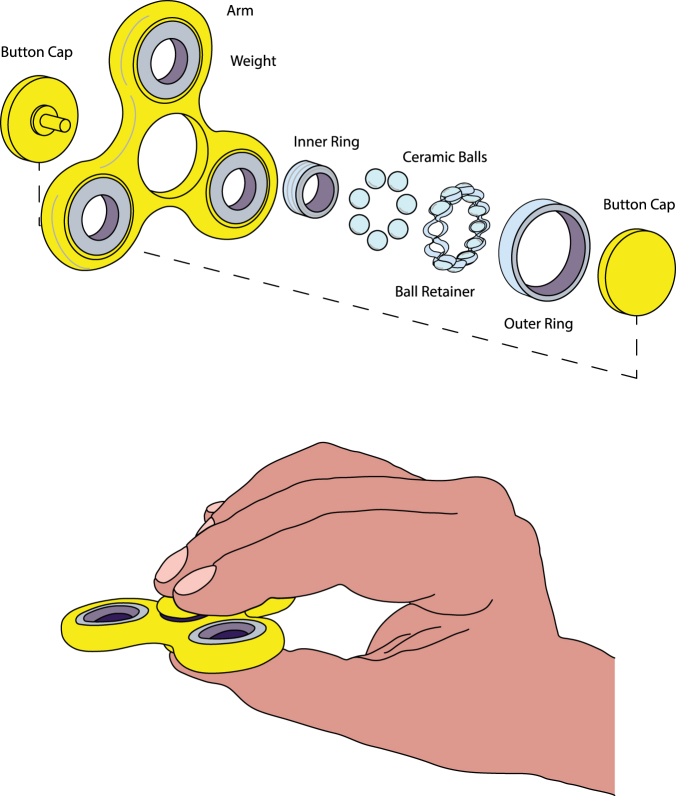
A diagram of Fidget Spinner and grip. In the upper panel it is possible to see the components of the fidget spinner, including the ball-bearing mechanism formed by the two rings interposed by ceramic balls, held together by a retainer. The bottom panel illustrates the way that the fidget spinner was asked to be held during the experiment, with the index and third fingers at the top and thumb at the bottom.

Fidget spinners are increasing in popularity and, as such, ambiguities regarding their possible effects are emerging. The mechanism behind the spinners is relatively simple. As any spinning apparatus, fidget spinners rotate around a central axis, formed by two rings. By using a ball bearing mechanism instead of simple sliding between the rings, friction may be reduced significantly during rotation. In order to further increase the duration of the rotation, fidget spinners are equipped with three wings (for most spinners) bearing weight distributed equally from the center. This allows to increase the moment of inertia of the spinner and, when an external force (or torque) is applied, results in a rotation that may last for a few minutes (Fig. 1). Also, by having the wings distant from the center of rotation, as for any lever-based system, less force is needed to induce a sustained rotation.

It is somewhat surprising that such a simple toy is subject to such a huge controversy, anecdotally as shown by social media. This controversy stems from the fact that fidget spinners are being currently marketed as devices that may help in increasing focus and attention, as well as general stress relievers. While some support these claims, e.g.^1^, other believe that fidget spinners are just a toy and, as such, do not possess any beneficial potential, e.g.^2^. Also, fidget spinners are considered to be a source of distraction in classrooms and are currently being banned in some schools throughout the United States^1,2^. Either way, these anecdotal beneficial claims regarding the spinners have rendered them an attractive mean for children suffering from ADHD and autism, as well as “focus enhancing” devices to the general population.

It should be mentioned however that, though anecdotal, these claims are not completely devoid of a scientific base as hyperactivity is often associated with some form of fidgeting and restlessness^3^. Therefore, the assumption that an external device may in some way attenuate hyperactivity and, consequently, maintain the attention seems reasonable. In fact, there are a few studies that have investigated the relationship between attention and fidgeting. Carriere and colleagues^4^ have investigated the relationship between fidgeting and mind wandering (i.e., presence of thoughts unrelated to the current task or decoupled from the external environment^5^), finding a strong association between the two, with increase in fidgeting behavior as attention decreases. They have concluded with the hypothesis that fidgeting increases the moment mind wandering takes place. Also, an earlier study found that when questioned, students believe that fidgeting is one of the strongest indicators for reduced attention^6^. Therefore, a close relationship seems to exist between fidgeting and attention, which was shown to be a function of time^7^.

More recent studies have further investigated the relationship between fidgeting and attention, trying to evaluate whether fidgeting in itself may modulate attention or only represents a manifestation of its reduction. In fact, fidgeting appears to play a role when cognitive tasks are to be performed. In two separate studies, children with hyperactivity were asked to perform cognitive tasks while their level of activity was being monitored^8,9^. In both studies, a positive correlation was found between the level of activity and task performance, suggesting that fidgeting plays a role in maintaining attention, in hyperactive children but not in typically developed children. These studies suggested that fidgeting may represent a compensatory mechanism for modulating attention and alertness, as well as augmenting CNS arousal during challenging tasks. This hypothesis is based on a model of ADHD according to which individuals exhibit a decreased tonic firing of the locus coeruleus-norepinephrine system, which would result in decreased cortical arousal and poor attention performance^10^. Under this view, an increase in activity, such as fidgeting, in individuals with ADHD may stimulate the system and, consequently, increase arousal^9,11,12^. Optimal arousal levels were shown to be necessary to maintain attention, and it was suggested that under attentional demanding conditions the level of stimulation could be modulated to optimize cortical excitability^13^. Further supporting the hypothesis that fidgeting could indeed represent a mechanism employed by individual with ADHD for maintaining attention by optimizing the level of arousal.

While fidgeting is characterized as the generation of small movements, many forms of fidgeting involve also the manipulation of external objects and seem to represent an important part of our day-to-day lives. As such, studies relative to fidgeting with objects like stress balls^14^, and doodling^15^ have emerged, further demonstrating the positive effect of fidgeting on both attention and concentration. In fact, the benefit of fidgeting activities has led to the design of workspaces for human-computer-interface with enough stimuli to favor fidgeting and, therefore, maintain attention while working^16^.

It should be mentioned that fidgeting was suggested to have also a stress-based origin, as most of the settings in which fidgeting was studied (i.e., those requiring sustained attention) may also be interpreted as stressful^7^. Moreover, in some people fidgeting appears to mediate the experience of perceived stress^17,18^. According to this view, fidgeting, intended as a manifestation of stress, would be expected to reduce performance in cognitive tasks^7^. However, this assumption remains as only a speculation for the moment.

When it comes to fidget spinners specifically, it is therefore plausible to assume that the manipulation of these toys may indeed help to increase concentration and attention. However, this is not necessarily achieved by merit of some intrinsic property the spinners themselves as fidgeting, in general, may have this beneficial effect. A different aspect of the spinners may be even more intriguing, seeing that their manipulation requires some level of control and coordination, especially when attempting to balance them as demonstrated by social media. Also, it is known that games in general, and specifically those requiring fine manipulation (e.g., video games), may improve coordination, precision and dexterity (e.g.^19,20^). As such, a plausible assumption is that the same may also be accomplished by fidget spinners. Especially when considered that repeated usage of these fidget spinners may render their manipulation automatic. The same as in practice, where the performance of a task eventually becomes second nature as a function of practice. It is well established that practice improves motor performance both in a task specific manner as well as by means of skill transfer (i.e., practice of certain type of task may improve performance in different tasks that rely on the same type of control)^21^. Therefore, fidget spinner manipulation may enhance fine motor control and, seeing that these objects are widely used, they have an added value of improving motor control in a population-based manner. Also, fidget spinners are generally perceived as an enjoyable pastime and thus, adherence is more likely.

It is evident that the spinners possess also some vibratory component to them, which we quantified according to Discrete Fourier Transform magnitude showing the principal component at a frequency of about 10 Hz (although magnitude is likely to vary between different spinners, see Methods). There are various studies that demonstrate that vibration may also affect motor control (e.g.^22^). Taken together, perhaps the combination of these factors, repeated manipulation and vibration, may in fact be favorable for promoting precision in fine motor control.

## Materials and Methods

### Participants

Eighty-one healthy adults were recruited for this study (age: 23.51 ± 2.47 years; 29 males). All participants were right handed (83.64 ± 13.91; laterality score from the Edinburgh Handedness Inventory^23^); they were naive to the task and the purpose of the study. All participants were free of documented visual, motor, neurological impairments. The participants were university students who volunteered for the study. Participants were not paid for their participation. The study protocol was approved by the Institutional Ethics Committee (Comitato Etico Area Vasta Centro AOUCareggi, Florence, Italy; Prot. N. 2015/0018234, Rif. 63/12) and all procedures conformed to the code of ethics of the Declaration of Helsinki. All participants gave written informed consent.

### Task and Set up

**Figure 2:**
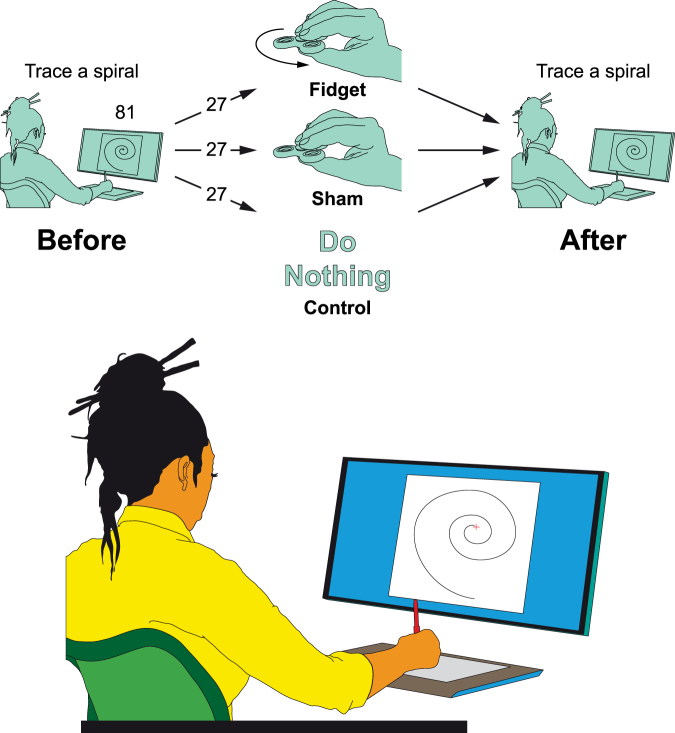
Experimental design. The upper panel illustrates the experimental design for this study. All participants were initially asked to trace a spiral (i.e., Before trial). After the first tracing, participants were divided into one of the three groups: Fidget, Sham or Control, and were asked to either rotate the spinner (i.e., Fidget group), hold the spinner (i.e., Sham group) or do nothing (i.e., Control group), for one minute, followed by a second tracing of the spiral (i.e., After trial). The bottom panel illustrates the working station used for the tracing. The graphic pen tablet was placed in front of the screen, and the participants were asked to trace the spiral, while seated, without the support of either wrist, arm, or elbow, in such a way that the only contact was made through the pen on the tablet.

Fine motor control can be tested with spiral drawing that offers a reliable -on the fly- measurement; in addition, by digitizing the procedure, a quantitative objective assessment may be obtained (e.g.^24–28^). As a quantitative measure, we used a spiral-tracing task to assess for fine motor control before and after using a fidget spinner (i.e., Fidget group), and compared these results to those obtained from a Sham and Control groups (Fig. 2).

Each participant was tested individually. Participants were randomly assigned to either a Control, Sham or Fidget group (27 participants per group; Fidget −10 males, Sham −8 males, Control −11 males). Before and after each trial, participants were asked to trace a spiral, beginning from the center and going outward, using graphic pen tablet (Wacom Intuos^®^ CTH-690AK, Tokyo, Japan; active area: 216 × 135 mm; Fig. 2).

To exclude performance differences between genders, the results of the tracings were evaluated by using an unpaired two sample t-test, not revealing any significant differences between genders, independently of group, for both first and second tracings, and also within groups for the second tracing.

The spiral templates were designed for a medial to lateral performance of the dominant hand (e.g., counter-clockwise for the right hand). The participants were instructed to trace the spiral while seated without the support of either wrist, arm, or elbow, in such a way that the only contact was made through the pen on the tablet (Fig. 2). We also specified to trace the spirals as precisely as possible with no regard to the speed of execution.

For the Fidget group, the trial consisted of rotating the fidget spinner, placed in the dominant hand. Participants were asked to hold the spinner with their thumb, index and third finger and to maintain the spinner horizontal to ground having the thumb placed at the bottom, and the index and third finger placed at the top of the spinner (Fig. 1). The reason for maintaining this horizontal position is that this way gravitational forces are equally distributed through the wings of the spinner. Once rotation was initiated, participants were asked to maintain this position for one minute, timed by a stopwatch. For the Sham group, participants were asked to hold the spinner in the same way as for the Fidget group without inducing a rotation and to maintain this position for one minute. For the Control group, participants were asked to do nothing for one minute.

### Analysis

**Figure 3:**
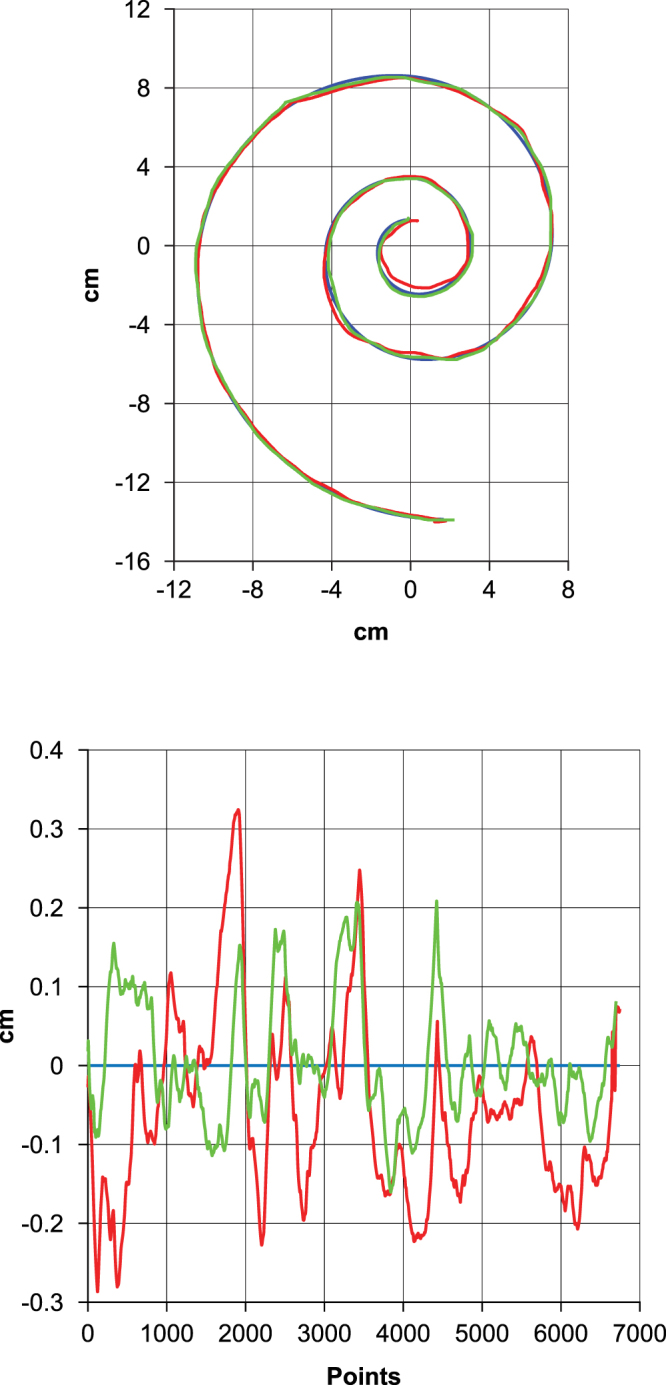
Spiral analysis. An example of how the spirals were analyzed. In the upper panel, it is possible to see three spirals that correspond to the template (blue), tracing in the Before trial (red), and tracing in the After trial (green). It should be noted that the first tracing was not visible to the participant during the second tracing. In the bottom panel it is possible to see the differences between the tracings more clearly, with the template corresponding to zero, and the tracings as deviations from the template. In this example it is possible to see an improvement in the After trial (green) compared to the Before trial (red). The participant in this case was part of the Fidget group, with a mean RD of 0.13 cm and a total area of deviation of 3.14 cm^2^ in the Before trial and 0.09 cm and 2.19 cm^2^ in the After trial.

We have developed an algorithm using Matlab for spiral analysis. The algorithm consists of a serial angle-based calculation of the traced spiral deviations from the template. Points of the tracing (n = 6,643 per traced spiral, normalized to the size of the template) were organized both according to their distance from the spiral center as well as according to the angle. For each point the residual difference (RD) between the tracing and the template was measured, considering the template as the expected value (Fig. 3). Since we are interested only in deviations from the template, RDs were considered as absolute values. For each tracing the mean RD and total area of deviation (considered as the area between the template and the drawn spiral) were calculated.

Quantification of the fidget spinner vibratory component was made with an accelerometer (ADXL330, Analog Devices Inc., Norwood, MA, USA), sensor output was acquired and digitized at 200 Hz through PCI-6071E (12-Bit E Series Multifunction DAQ, National Instruments, Austin, TX, USA) and analyzed with Matlab according to Discrete Fourier Transform.

### Statistics

The mean and standard deviation, per group and per trial, were calculated for both mean RD and total area of deviation. A two-way ANOVA was implemented to evaluate the differences, in mean RD and total area, both between groups (i.e., factor 1: Fidget, Sham, and Control) and between trials (i.e., factor 2: Before and After trials). ANOVA analyses were followed by a Bonferroni post-hoc test to confirm the significance of the differences between groups and between trials. Furthermore, the root-mean-square standardized effect, namely Ψ, was calculated as the effect size estimator for ANOVA analysis, which was used as the effect size for power analysis calculation^29^. Statistical power was calculated using G*power 3.1.9 with an α value of 0.05^30^.

### Data Availability

All data generated or analysed during this study are included in this published article (and its Supplementary Information files).

## Results

**Figure 4:**
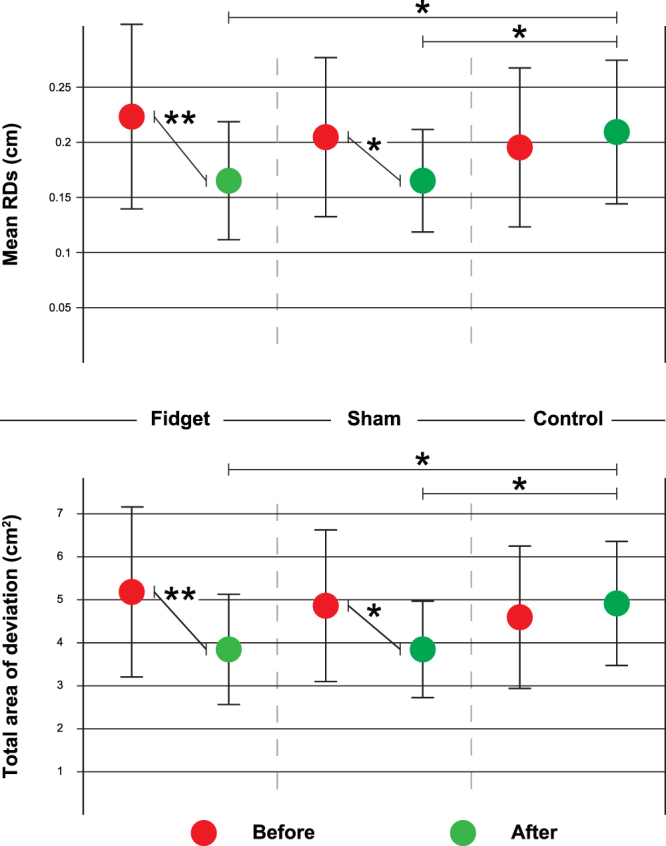
Results. Circles represent the group mean and vertical bars indicate the standard deviation. Circles are color coded according to trial (red for Before, green for After). It is possible to see that for both Fidget and Sham groups there is a significant improvement in the After trial with p-values <0.01 (**) for the Fidget group and <0.05 (*) for the Sham group for both mean RD (upper panel) and total area of deviation (lower panel). Also, it is possible to see a significant difference in the After trial, for both mean RD and total area of deviation, between Fidget and Control groups and Sham and Control groups with p-values <0.05 (*), but not between Fidget and Sham groups.

Based on both mean RD and total area of deviation from the template (Fig. 3), there seems to be a general improvement in the After trial for both Fidget and Sham groups but not for the Control group (Fig. 4). For the Fidget group, mean RD improved from 0.22 ± 0.08 cm to 0.16 ± 0.05 cm; total area from 5.22 ± 1.95 cm^2^ to 3.84 ± 1.27 cm^2^. For the Sham group, mean RD improved from 0.20 ± 0.07 cm to 0.16 ± 0.04 cm; total area from 4.85 ± 1.78 cm^2^ to 3.85 ± 1.12 cm^2^. For the Control group no improvement was found, with mean RD of 0.19 ± 0.07 cm Before and 0.21 ± 0.06 cm After; total area measured 4.60 ± 1.66 cm^2^ and 4.92 ± 1.49 cm^2^, respectively.

ANOVA analyses preformed on both mean RD and total area of deviation confirmed a significant difference between trials (i.e., factor 2: Before and After; F-values: 7.37 for mean RD and 7.71 for total area, d.f.:1), with p-values of 0.007 (for mean RD) and 0.006 (for total area). Specifically, the within group results for both Fidget and Sham groups were found to be statistically significant for both mean RD (p-values of 0.001 and 0.026, respectively) and total area of deviation (p-values of 0.001 and 0.020, respectively). These results were also confirmed by the Bonferroni post-hoc test. Contrary, the within group results for the Control group were not found to be significantly different between trials (p-values of 0.455 for mean RD and 0.458 for total area; Fig. 4).

When analyzing the results between groups (i.e., factor 1), no differences between groups were found for the Before trial (i.e., first tracing), with p-values all above 0.05 for both mean RD (p-values of 0.99 for Fidget vs Sham, 1 for Sham vs Control, and 0.41 for Fidget vs Control) and total area (p-values of 1 for Fidget vs Sham and for Sham vs Control, and 0.44 for Fidget vs Control) according to the Bonferroni post-hoc test. However, when analyzing the After trial, significant differences were found between both Fidget vs Control (p-values 0.042 for mean RD and 0.039 for total area of deviation) and Sham vs Control groups (p-values 0.043 for mean RD and 0.041 for total area of deviation). No statistical differences were found between the Fidget and Sham groups for the After trial (p-values of 1 for both mean RD and total area of deviation). The statistical power relative to the sample in this study was found to be 87.7%, with a calculated effect size estimator Ψ value of 0.389^29^.

## Discussion

Our results suggest that the use of fidget spinners may indeed better fine motor control to a certain extent, as shown by the within group analysis. However, it would be imprudent of us not to consider the fact that this general improvement was also evident for the Sham group. Taken together, these results suggest that fine motor control may be related more to the general manipulation of objects and not necessarily inherent to fidget spinners themselves. Considering our sample of young healthy subjects, our results should apply to all healthy subjects. It is possible, that in certain special groups, such as that of ADHD, the effects could be even greater, seeing that fidgeting in general was already shown to have a beneficial effect on this type of population. However, this remains as a mere speculation for the moment that may be elucidated by future studies.

The observed improvement in performance for the Fidget and Sham groups may be due to an additional attentional component on the motor effector related to handling of the fidget spinner between trials. This may explain why improvements were found in both Fidget and Sham groups but not in the Control group. In fact, by examining dual task paradigms it is evident that while performing a motor task concurrent with a cognitive task, some modifications to the motor performance occur, suggesting that the any motor task, even when not fully concentrated, requires allocation of attentional resources for the performance^31–33^. Also, object manipulation specifically was shown to require the integration of sensory, motor and cognitive systems^34,35^. Moreover, it was shown that both internal and external focus may affect motor performance, with the latter being more effective in improving performance^36,37^. Therefore, it is possible that the handling of the fidget spinner between trials may contribute to divert the attention toward the handling hand (i.e., internal focus) or the fidget spinner itself (i.e., external focus). Consequently, a higher level of performance may be achieved faster and retained more effectively^38^.

Another possibility would be that while handling the object, the motor areas responsible for the movement during the task remain active. It was shown that activation of motor areas, even when no movement is occurring (e.g., motor imagery), may influence performance^39,40^. Also, when developing motor skills, it was shown that executing different tasks may help in improving a specific skill by means of skill transfer when the same effectors are used^21,41^. Under this view, the handling of the fidget spinner between trials, by keeping the same motor areas activated, may be comparable to practice by means of skill transfer and, therefore, improve performance in successive trials. A general improvement due to trial repetition may be excluded seeing that the Control group did not demonstrate an improvement between trials. An fMRI study could provide information relative to this hypothesis. Also, it would be interesting to evaluate the amount of attention allocated during the use of fidget spinners by a dual task paradigm. Perhaps by doing this, the effect would be better characterized and, consequently, also controlled.

In our study we tried to obtain a homogeneous group of individuals, all being young adults free from any reported neurological or visual impairments that may interfere with the task. However, there are other variables that may influence motor performance that were not evaluated in this study. It was shown that motor and cardiovascular fitness as well as academic skills are related and could influence one another^42^. Also, motivation was shown to be a determining factor for success when developing motor skills^21^. Therefore, it is possible that by evaluating and/or controlling these variables some specific correlations may emerge. We assume that by our choice of the sample, the motor fitness, motivation, and age would be relatively similar among subjects, and therefore, would not affect the present research. However, it would be interesting to test if the effect reported here would also be present in other types of groups with different motor and cardiovascular fitness (e.g., professional athletes vs sedentary individuals) and age (e.g., children vs adults).

It should also be considered, that our study was concentrated only on the immediate effect and, therefore, we cannot predict whether these effects are also long lasting. While this remains as an open question, it is also true that the continuous manipulation of objects may eventually better dexterity^8,19–21,43^. In fact, when manipulating objects, the mechanical properties of both hand and object must be accounted for. This is made simple for a rigid object that is held firmly in the hand, as movement of the object is equivalent to controlling the movement of the hand whereas for non-rigid objects, movement of the object is made by the interaction between hand and internal dynamics of the object^44^. The manipulation of unknown objects (i.e., unknown dynamics) is made by estimation of either the dynamics of the object or employment of different strategies for control, both of which are based on past experiences^44^. Therefore, it is possible to assume that the longer and more varied is the manipulation of objects, the easier would be the successive manipulation of new objects. This way, continuous manipulation will add to the repertoire of experiences and strategies for future human-object interactions, especially when considering that experience is a determining factor for the success of a planned motor response^45^. Further supporting this notion is the fact that when the physical properties of the arm are altered by an object, the internal model of dynamics of the adapted arm to the new physical condition is maintained^46–48^. This type of adaptation may indeed influence the predictability of the object’s dynamics in future human-object interactions, which was suggested to be a primary criterion for strategy selection^49^. When these concepts are combined with skill transfer, it is probable that a continuous manipulation of objects may indeed influence dexterity and motor control. Examples for this can be found when examining the effects of video games as well as chopsticks on dexterity^19,20,50,51^. Therefore, it is possible that the repetitive manipulation of fidget spinners may influence motor control. Moreover, by being a toy that is considered to be enjoyable, fidget spinners may stimulate even more people to utilize them, much more efficiently than refine exercises aimed to improve fine motor control.

## Electronic supplementary material

Dataset
